# The experiences of family members in the nursing home to hospital transfer decision

**DOI:** 10.1186/s12877-016-0359-2

**Published:** 2016-11-15

**Authors:** Kathleen Abrahamson, Brittany Bernard, Lara Magnabosco, Arif Nazir, Kathleen T. Unroe

**Affiliations:** 1 Purdue University School of Nursing, 502 N. University Street, West Lafayette, Indiana 47907 USA; 2 Indiana University Center for Aging Research, Indianapolis, Indiana USA; 3 Regenstrief Institute, Inc, Indianapolis, Indiana USA; 4 Department of Medicine, Indiana University School of Medicine, Indianapolis, Indiana USA

## Abstract

**Background:**

The objective of this study was to better understand the experiences of family members in the nursing home to hospital transfer decision making process.

Semi-structured interviews were conducted with 20 family members who had recently been involved in a nursing home to hospital transfer decision.

**Results:**

Family members perceived themselves to play an advocacy role in their resident’s care and interview themes clustered within three over-arching categories: Family perception of the nursing home’s capacity to provide medical care: Resident and family choices; and issues at ‘hand-off’ and the hospital. Multiple sub-themes were also identified.

**Conclusions:**

Findings from this study contribute to knowledge surrounding the nursing home transfer decision by illuminating the experiences of family members in the transfer decision process.

## Background

Nursing home residents are at risk for complications surrounding hospital transfers [1]. Risks of hospitalization include infection, discontinuity of treatment or medication, miscommunication surrounding advance directives, immobility, delirium, and emotional distress [2, 3]. Some hospitalizations are necessary; however, many resident transfers from nursing home to hospital are thought to be avoidable [4–7]. Examples of avoidable hospitalization include cases where: a.) changes in condition could have been prevented; b.) intervention could have happened earlier to prevent decline; c.) the transfer is a result of communication breakdown between nursing staff and provider; d.) clinical care (e.g., Intravenous therapy or diagnostic testing) could potentially be available in the nursing home setting but was not provided; or e.) the transfer was inconsistent with resident goals of care. The Centers for Medicare and Medicaid Services (CMS) have reduced payments to hospitals with excess readmissions, and similar financial penalties for nursing homes are pending [8].

Each hospitalization of a nursing home resident is the end result of a decision making process that involves multiple stakeholder perspectives. Factors known to impact the hospitalization decision include provider availability, clinical capacity of the nursing facility, nursing perception of resident acuity, communication between nursing staff and provider, resident wishes, and desires of family members [9, 10]. Despite the availability of evidence supporting the role of communication between medical providers, nursing home staff, residents, and family members in the transfer outcome, little is known about family member input in to the decision making processes surrounding the transfer [11–13]. Existing qualitative studies of family member perspectives note an emphasis on resident dignity, personhood, values and desires; the strength of the relationship between family members and providers; communication of information from nursing home staff; and perceived timeliness of nursing home staff response to a resident concern [13, 14]. A study of provider perspectives of family member involvement in transfer decisions found that families may respond to condition changes as a crisis and are often emotionally unprepared and not provided with adequate clinical information from the nursing home to participate in clinical decisions [12].

A number of studies have viewed the hospitalization decision retrospectively through chart abstractions or claims data to determine if a hospitalization was potentially avoidable and what clinical factors may have impacted that decision. The current study examines family member thought processes in reference to actual transfer events of long stay nursing home residents with dementia. The transfer events were not analyzed to determine avoid-ability, but instead discussed with the involved family member to elicit their viewpoint and experiences surrounding participation in the transfer decision and process. The objective of the current study was to better understand the experiences of family members who serve as surrogate decision makers in the nursing home to hospital transfer decision making process.

## Methods

Data for this study were collected within the context of the CMS Center for Innovations- funded Optimizing Patient Transfers, Impacting Medical quality and Improving Symptoms: Transforming Institutional Care (OPTIMISTIC) project. One of seven sites for the CMS Initiative to Reduce Avoidable Hospitalizations of Nursing Facility Residents, OPTIMISTIC is a 4-year (2012–2016) demonstration project based at Indiana University aimed at the reduction of avoidable hospitalizations among long stay (>100 days) dually eligible (Medicare and Medicaid) residents. The OPTIMISTIC project includes 19 partner facilities and contains 3 core intervention components: improving medical care, enhancing transitional care, and access to palliative care. The primary intervention involves embedding a registered nurse (RN) in each facility to lead delivery of the project components, supported by project nurse practitioners (NPs) who provide care within a group of facilities [15]. Although the data for this project was gathered within facilities participating in the OPTIMISITC project, the aim of the current study remained separate, and data collected for this current analysis were independent of data collected for the larger OPTIMISTIC project.

Semi-structured interviews were completed by authors B.B. and L.M., project coordinator and research associate, with 20 family members of residents from within 9 nursing facilities who had experienced a hospital transfer within the past three months. Interview respondents were identified by the OPTIMISTIC project nurses within the facility as being family members highly involved in the transfer decision of a resident with dementia, i.e.,-who had acted in the role of surrogate decision maker. Prior to requesting an interview, the investigators also ascertained whether the family member was actively involved in the resident’s day to day care – defined as contact with the resident and/or nursing staff at least weekly, as determined by the resident’s unit nurse. In order to best capture transfer cases with adequate complexity for discussion, it was determined by the research team that cases were prioritized but not limited for interview based on symptoms at the time of transfer – specifically, transfers with a primary symptom of shortness of breath, confusion, or change in mental status were identified and contacted first. Other reasons for transfer included abdominal pain, behavioral symptoms, bloody stool, fever, malaise, and pain. The project coordinator communicated with OPTIMISTIC nurses within the facility to confirm eligibility and obtain contact information of involved family members. Prospective interviewees were then contacted by B.B. and asked to participate in the study. All identified family members agreed to participate in an interview. Participating family members were residents’ daughters (13), sisters (2), son (1), daughter in-law (1), niece (1), nephew (1), and wife (1). Interviews were semi-structured, approximately 30 min long, and recorded for de-identified transcription. Interview length was not pre-determined, and respondents were encouraged to provide details surrounding their experiences. The interview guide contained 26 base questions (ranging from ‘How often to you visit?’ to ‘Please describe the transfer?’) plus probes. Questions addressed the areas of communication with staff; decision making regarding resident care; and the transfer situation. Interviews were conducted over the phone between November 2014 and March 2015. Institutional Review Board approval was obtained prior to conducting interviews and participants were read a study information sheet during telephone contact. Informed consent was received from each interview respondent prior to initiation of the interview.

The method of analysis was similar to the Framework Method as described by Gale et al. [16] as an appropriate method to conduct qualitative content analysis within multidisciplinary research teams. Transcripts were analyzed independently but simultaneously over a one month period by 4 co-authors (KA, BB, LM and KU) to develop a list of thematic codes. Codes were not specified prior to analysis, but were allowed to emerge from the data instead. Discrepancies between authors were discussed during in-person meetings and refined until a final code list was developed. Transcripts where then re-analyzed simultaneously by the 4 co-authors (KA, BB, LM and KU) using the final code list. Interview respondents were not invited to check or review the code list. A matrix of identified codes was developed and codes were further grouped according to over-arching unifying themes, such as aspects of the transition process, nursing home factors, resident factors, the role of the MD, and family member factors. The themes were discussed further among co-authors prior to manuscript development.

## Results

Nearly all of the 20 family member interview respondents (19) described their role in the residents’ care in terms consistent with advocacy. At times this was a formal Power of Attorney arrangement, and other times respondents described themselves as “watching over” “knowing everything at all times” “call attention to the employees when he needs something” or “sticking by her”. The majority of the respondents (14) explicitly described having no relationship or contact with the resident’s physician or the nursing home medical director. Family members responded to a question asking about communication with a physician with phrases such as “I don’t think there is a doctor there ever” “I have never met a doctor” and “We don’t have a lot of contact with the physician that sees her. Most (information) comes through the nursing staff.” Nursing staff members were noted by all as the primary means of obtaining information on the residents’ care, either in person or by telephone, and the majority of respondents (16) described having confidence in the nursing home staff to provide information. Half of the family members (10) described caring for their family member as difficult or demanding for nursing home staff, and 5 respondents made comments indicating a perception that their family member was easy or not demanding of staff time. When asked to retrospectively reflect on whether the transfer of their family member was a good decision or if the hospitalization could have been avoided, most respondents expressed confidence in the decision to transfer and 1 respondent expressed regret with the decision due to the resident’s perception of poor care at the hospital.

Responses relating to factors which influenced the experiences of family members surrounding the hospitalization decision clustered around 3 separate but interrelated themes: family perception of nursing home capacity to provide needed care; resident and family choice; and issues surrounding ‘hand-off’ and hospital care. Within those themes multiple sub-themes were noted and are discussed below. Themes are also displayed in Table 1.

**Table 1:** Themes and Sub-themes from Family Member Interviews

Family perceptions of nursing home capacity to provide needed medical care
Perception of low level care and/or services in nursing home
Perception of lack of physician presence in nursing home
Perception that more care is available in the hospital
Belief that the nursing home does not notice changes quickly enough
Perception of staff member supporting transfer
Nursing home staff is familiar with resident (benefit)
Resident and family choices
Advance directives
Resident desire to remain in the nursing home
Quality of life
The challenge of end of life decisions
Issues at hand-off and at hospital
Poor communication between nursing home and hospital
Low satisfaction with hospital care

### Perception of nursing home capacity to provide medical care

Family members described a perception that more medical care would be available in the hospital, and that nursing home staff may not attend to changes in condition quickly enough. Some respondents based this perception on past instances where a change in condition resulted in a poor outcome, others on a desire for care that is more curative, physician-driven, or technologically advanced than what they see as available in the nursing home. Family members did not generally phrase this as a dis-satisfaction with the nursing home for daily caregiving, but instead as a perception that limited medical care was available in the nursing home setting when problems arose. Family members made statements such as:

*They can get a lot of what I call comfort care and basic pills dispensed and watched over and things like that but you can’t get medical care.* (Respondent C, daughter)

*I felt like they were doing as much as they could. They were short staffed on Christmas Holiday and trying to do the best they could.*

(Respondent G, daughter)

Some family members mentioned specific procedures such as swallowing studies, placement of gastric tubes, and intravenous antibiotics; others spoke more generally about hospital care “making more improvement” and needing the hospitalization to “get her back on track”. Timeliness of the nursing home response to conditions changes emerged as a particular concern of family members. Respondents expressed that nursing home staff did not address changes in condition quickly enough to avoid hospitalization and/or should have identified a need for hospitalization earlier. Particular situations noted by family members included waiting to transfer until after the weekend, the lack of timely diagnostic testing such as an X-ray, and not quickly linking subtle changes in mood or mental status to a change in health status.

However, respondents also discussed feeling that staff familiarity with their resident was a benefit of remaining in the nursing home and avoiding hospitalization. The opinions of staff regarding the need for hospitalization appeared to have influence on family thinking surrounding the need for transfer, and respondents spoke positively regarding the importance of the relationships between staff members and their loved one.

*The nurses and respiratory therapists that are with him every second of the day, they know what he needs and they have just grown to catch the illness earlier. They just know how to take care of him.*

(Respondent B, daughter)

*They (staff) are very in-tune to his situation…They were giving me their recommendations…obviously I said absolutely, let’s get him to the hospital.*

(Respondent Q, brother)

### Resident and family choices

Perhaps not surprisingly, resident wishes and desires emerged as important to family members who had participated in a hospitalization decision. When asked about advance care planning, nearly all mentioned advance directives or discussion of advance care planning that had taken place prior to the hospitalization decision. When asked if the presence or absence of advance directives had impacted the family member’s decision to advocate for hospitalization, most indicated that it did not. The primary reason given for not giving greater consideration to advance directives was that the reason for hospitalization was viewed as non-life threatening and the advance directive was not seen by the family member as applicable to the situation.

Some family members discussed the challenge of making the hospitalization decision when their family member prefers not to leave the nursing home, i.e.,-overriding resident wishes.

*He would rather stay at the nursing home when he is sick. He does not want to go to the hospital. So, I think that does (influence my decision) a bit.*

(Respondent B, daughter)

*She doesn’t want to go to the hospital. Even if she knows she has to, she just doesn’t want to go. I have to be in charge of that (decision to hospitalize).*

(Respondent S, niece)

Additionally, family members discussed the importance of maintaining resident quality of life and not wanting medical treatment that would result in discomfort with little benefit.

*I don’t think anything is going to improve her health. I think relief of her pain and any confidence she might feel in her caregivers there (in the nursing home) would be the only difference that could be made.*

(Respondent D, daughter-in-law)

Family members described the end-of-life choices as a particularly challenging aspect of their role. When they perceived that little could be done to improve the resident’s health, the decision to transfer to an acute care setting resulted in ambiguity or uncertainty concerning the value of the intervention.

*They said they could just keep him comfortable in the nursing home, and make him a hospice patient. We opted to send him to the hospital to have antibiotics. Because he was up walking around…he was able to have a little quality of life so I figured he may have a few more years left, maybe he had some quality of life left.*

(Respondent E, niece)

### Issues at ‘hand-off’ and the hospital

When asked to describe their experience at the point of transfer, family members often described the challenge of maintaining the resident’s wishes or preferences for care in the hospital setting. Some respondents relayed this challenge as a perception that communication between the hospital and the nursing home was poor and disjointed. Some respondents also reflected that the decision to transfer was a good one, even in situations where they described hospital care as poor quality. Issues raised by family members included lack of communication with family members, unnecessary changes in medications, and lack of attention to the needs of aged patients with cognitive or functional impairments. Communication between the nursing home and the hospital appeared to be of primary concern to respondents.

*The staff here at the nursing home said that when she got to the hospital they should have had all her information. And obviously it got lost between the nursing home and the hospital, which is a half block. 100 feet. It is not like they go a long distance.*

(Respondent S, niece)

*(The hospital) weren’t giving her the meds that they were giving her at the nursing home for Alzheimer’s and stuff, and…well, she was getting violent. I kept asking and they said ‘yea’ but it took 4 days to get the medication she was on in the nursing home.*

(Respondent G, daughter)

## Discussion

Family members have been described by providers and nursing home staff as being an important driver in the nursing home to hospital transfer decision [9, 11]. Yet, little research has specifically focused upon the experiences of family members who have participated in the decision making process. The objective of this study was to obtain in-depth information regarding the experiences of family members, through the voices of family members who had very recently participated in a transfer decision. The current study contributes to knowledge surrounding the nursing home to hospital transfer decision by focusing on the family’s experience as the transfer decision is made.

Families generally did not perceive a physician presence in the nursing home, a theme which has emerged in prior literature addressing factors that impact hospitalization from provider and nursing administrator perspectives [9, 17]. Our findings also indicated that families perceived nursing homes to provide a low-level of medical care, despite a view that nursing home staff knew their family member well. Previous research indicates that families perceive changes in condition to be a crisis [12] and place importance on the relationship with their loved ones medical provider [14]. As such, increased physician presence has been associated with low hospitalization rates [17]. One method to increase medical provider presence is the use of advanced nurse practitioners (NPs) to provide assessment and treatment to nursing home residents at risk for hospitalization. Programs such as Evercare [18] have demonstrated the effectiveness of NPs in reducing nursing home resident hospitalizations, and interventions to increase the presence of medical providers within nursing homes may have a positive effect on family member confidence in the ability of the nursing home staff to manage their loved one’s condition. The OPTIMISTIC intervention includes NPs that provide as needed coverage in the partner facilities but it is possible that a higher level of presence will be needed to change family perceptions of physician/ NP engagement.

Most of our respondents noted either having advance directives in place or having had a conversation with a provider regarding resident treatment preferences. Although hospitalizations occur frequently near the end of life, the presence of advance directives was not noted by respondents to be key in the hospitalization decisions. Treatments for an acute change in status apart from what family considered being ‘end of life’ appeared to be considered separately from any previous discussion surrounding life-sustaining measures. Respondents also discussed the trust and importance they placed on information arising from the nursing staff. Further exploration into nurse to family member communication surrounding the nature of the condition change and the risks/benefits of hospitalization would be beneficial. The use of structured tools to record preferences for medical care such as POLST forms (polst.org) includes preferences regarding hospital transfer. Use of POLST has been shown to reduce avoidable hospital transfers of nursing home residents [19].

There is evidence within our study that many hospitalization decisions were influenced by family member perception of the timeliness and efficacy of clinical intervention within the nursing home. Perception of clinical capacity may not reflect actual capacity; in the current study families gave little concrete evidence to back perceptions that the resident would be better cared for in an acute setting. While family members made statements that more or better medical care could be provided in a hospital setting, many also reported negative experiences regarding the care of hospitalized family members. An underlying thread in findings is that family members are left to make decisions based upon trust, relationship and perceived accuracy of information, all of which can be influenced by nursing home culture and environment. Communication and relationship building between family members and providers, including direct care nursing staff, has potential to reduce costly avoidable hospitalizations. Additionally, timely and proactive education of family members on availability of licensed on-call clinical teams and the ability of nursing home staff to manage medical needs and the risks of hospitalizations, may assist family members to make the decision most congruent with the wishes of the resident.

Family members are important decision-makers when they are present and involved, but their role in a given clinical decision is variable depending upon interest, knowledge and availability. Family members can be an important link to resident history and desires, especially in cases where residents are unable to communicate such wishes for themselves. This voice of resident advocacy is particularly important as nursing homes seek to provide care that is individualized and increasingly person-centered. However, it is important that family members be considered as partners in decision-making. Our previous research has noted the challenges faced by nurses as they span the boundary between various stakeholders to negotiate the hospitalization decision [20]. Nursing staff require time to communicate with families and appropriate clinical support in order to care for sick residents in place. Additionally, nursing home staff members would benefit from training specific to the experiences of family members and appropriate communication surrounding the medical needs of the resident. Our data demonstrates that family members value resident comfort and quality of life, areas of nursing home care that can be emphasized by nursing home staff, often without hospital level medical intervention.

There are a number of limitations to this study. First, the study took place within a context that was actively seeking to reduce avoidable hospitalizations and may not reflect the experiences of family members within communities without such efforts, potentially reducing the transferability of these findings. Second, our study is relatively small sample and additional work is needed to go beyond these preliminary findings to increase the authenticity and range of findings by going deeper into family member perspectives. The study only sought the perspectives of family members for scenarios where a transfer occurred and it is quite possible that family members’ perspectives around decision-making where a hospitalization was avoided will reveal different themes and roles. Although the aim of this research was to ascertain in-depth information, additional research that more extensively examines family member satisfaction with the transfer decision, the role of family dynamics in decision making processes, and decisions surrounding end-of-life care would enhance the current literature. Future studies are needed to further delineate family roles in helping prevent avoidable hospitalizations. Finally, nursing home quality and care processes impact outcomes and may lead to varying family/resident experiences. Future work needs to explore impact of facility quality on the transfer decision.

## Conclusions

Implications of this research include family members lack of direct contact with physicians within nursing homes, the role of nursing staff as key communicators of clinical information and partners in decisions regarding transfers, the importance of advocacy to family members when considering care alternatives, and the potential to educate family members about the level of clinical care possible within the nursing home environment. Future interventions may benefit from focus on more timely and precise communication with family members, as this appears to be an area of particular salience for family members who actively participate in resident care decisions.

## Abbreviations

CMS: Centers for Medicare and Medicaid Services
NP: Nurse practitioner
OPTIMSITIC: Optimizing patient transfers, impacting medical quality and improving symptoms, transforming institutional care
POSLT: Physician orders for life-sustaining treatment
RN: Registered nurse
